# Boredom, emotional dysregulation and avoidance coping strategies: Which is their role in youth mood disorders?

**DOI:** 10.1192/j.eurpsy.2023.1500

**Published:** 2023-07-19

**Authors:** A. Cicolini, L. Orsolini, G. Longo, M. Silvestrini, U. Volpe

**Affiliations:** 1 DIMSC; 2 Polytechnic University of Marche, Ancona, Italy

## Abstract

**Introduction:**

Coping and emotional regulation mechanisms may play a significant role in the neurodevelopment and in the subsequent psychopathological trajectories, especially in youth. The boredom dimension may also have a pathoplastic role.

**Objectives:**

Considering the poor literature in adolescents and young people (15-24 years-old), our study aims at investigating the relationships between coping patterns and emotional dysregulation as well the mediatory role of boredom, by particularly focussing on a sample at early onset of mood disorders.

**Methods:**

Cross-sectional, observational design study. Descriptive analyses were performed considering a set of socio-demographic and clinical variables (DERS, MSBS, COPE-NVI). Kruskal-Wallis, Spearman correlations and linear regression models were performed between DERS (dependent variable) and COPE-NVI (independent variable), together with mediation analyses (MSBS as mediator).

**Results:**

86 subjects (mean age=18.4±2.8) were enrolled. DERS score was 114.8±33.3, COPE-NVI was 129.1±22.1, MSBS was 136.22±45.8. Positive correlation between DERS total and *avoidance strategies* (r=+0.6,p<0.001) and negative correlation between DERS total and *problem orientation* strategies (r=-0.467,p=0.023) were found. Linear regression analyses showed statistically significant differences between DERS and COPE-NVI *avoidance strategies* (p<0.001) and COPE-NVI *problem orientation* (p<0.023). Mediation analyses confirmed the mediatory role of boredom dimension in the association between COPE-NVI *avoidance* subscale and DERS total (B=0.6849, p <0,001), between *avoidance* subscale and DERS *lack of acceptance* subscale (B=0.1286, p<0.001). Moreover, a mediatory role of MSBS inattention subscale was found in the association between COPE-NVI *avoidance* subscale and DERS *lack of control* subscale (B=0.1027, p<0.001).

**Conclusions:**

Maladaptive coping strategies (particularly avoidance) were associated with increased DERS levels. A predominant use of more adaptive coping strategies (i.e., problem solving, planning) were associated with lower DERS levels. Their relationship appears to be mediated by boredom dimension.

**Disclosure of Interest:**

None Declared

